# Special Section Guest Editorial: Special series guest editorial: Biomedical Imaging and Sensing III

**DOI:** 10.1117/1.JBO.26.3.033701

**Published:** 2021-03-13

**Authors:** Toyohiko Yatagai, Osamu Matoba, Yoshihisa Aizu, Yasuhiro Awatsuji, Yuan Luo

**Affiliations:** a Utsunomiya University, Utsunomiya, Japan; b Kobe University, Kobe, Japan; c Muroran Institute of Technology, Muroran, Japan; d Kyoto Institute of Technology, Kyoto, Japan; e National Taiwan University, Taipei, Taiwan

## Abstract

Guest editors Toyohiko Yatagai, Osamu Matoba, Yoshihisa Aizu, Yasuhiro Awatsuji, and Yuan Luo introduce the articles in the Special Series on Biomedical Imaging and Sensing.

In biomedical optics and photonics, optical tools are employed for the understanding and treatment of diseases, from the cellular level to macroscopic applications. At the cellular level, highly precise laser applications allow the manipulation, operation, or stimulation of cells, even in living organisms or animals. Optical microscopy has been revolutionized by a thorough understanding of the different markers and their switching behavior. Marker-free microscopy technologies, like CARS, SHG or THG-microscopy, OCT, and Digital Holographic Microscopy (DHM), are spreading into multiple biological and clinical imaging applications. OCT is continuously broadening its clinical applicability by becoming even higher resolution, higher speed and more compact. In the broader field of optics and photonics, biomedical imaging and sensing are the most quickly progressing and expanding areas. Techniques developed in these areas could greatly advance physical, engineering, and biological knowledge as well as optics and photonics technology.

The *Journal of Biomedical Optics* (JBO) special series entitled “Biomedical Imaging and Sensing” provides a collection of papers related to the above-mentioned topics, which are central to the annual Biomedical Imaging and Sensing Conference (BISC). The conference aims to provide an international forum for reporting recent progress in imaging and sensing technologies in the fields of biology and medicine, as well as related areas.

BISC includes a range of research, from basic cellular level research through clinical applications of various optical technologies. Topics cover medical and biological imaging instrumentation and techniques, advanced microscopy, advanced endoscopy, super resolution in biomedical imaging and sensing, computational imaging in biomedical imaging and sensing, adaptive optics in biomedical imaging and sensing, structured illumination in biomedical imaging and sensing, interferometry and holography in biology and medicine, optical coherence tomography, digital holography, quantitative phase imaging, diffuse spectroscopy and tomography, photoacoustic imaging, multimodal imaging and sensing, optical biopsy, multispectral imaging and sensing, spectroscopic imaging and sensing, scattering imaging, fluorescence imaging, molecular imaging, terahertz sensing, imaging and sensing techniques for biomedicine, optical fibers and sensors for biomedicine, multimodality optical diagnostic systems. Recently developed computational imaging techniques such as light field imaging, digital holography, and compressive sensing are expected to improve the image quality, deriving multimodal physical parameters from sparse information and overcoming weak light conditions by using photon-counting devices.

Although BISC’20 was canceled due to COVID-19 restrictions, the submitted papers are effective. A total of 8 papers were accepted for publication in JBO for this, the 3rd annual BISC-related special section, published as a series from December 2020 to March 2021. The papers included in this special section report recent research on the following topics: OCT with deep learning, differential interference contrast microscopes for deep tissue, multiplane phase contrast imaging and multiplane two-photon fluorescence imaging using a volume hologram, computational microscopy using parallel-phase-shifting digital holography, aberration-free microscopy using wavefront sensing by image-based correlation, and USB capsule endoscope. The following papers are included:

“High signal-to-noise ratio reconstruction of low bit-depth optical coherence tomography using deep learning” by Qiangjiang Hao, Kang Zhou, Jianlong Yang, Yan Hu, Zhengjie Chai, Yuhui Ma, Gangjun Liu, Yitian Zhao, Shenghua Gao, and Jiang Liu. https://doi.org/10.1117/1.JBO.25.12.123702
“Minimizing scattering-induced phase errors in differential interference contrast microscopy” by Wataru Takano, Shuhei Shibata, Nathan Hagen, Masaru Matsuda, and Yukitoshi Otani. https://doi.org/10.1117/1.JBO.25.12.123703
“Multiplane differential phase contrast imaging using asymmetric illumination in volume holographic microscopy” by Yu-Hsin Chia, Sunil Vyas, Jui-Chang Tsai, Yi-You Huang, J. Andrew Yeh, and Yuan Luo. https://doi.org/10.1117/1.JBO.25.12.123704
“Two-photon fluorescence imaging of subsurface tissue structures with volume holographic microscopy” by Xiaomin Zhai, Sunil Vyas, J. Andrew Yeh, and Yuan Luo. https://doi.org/10.1117/1.JBO.25.12.123705
“Modularized microscope based on parallel phase-shifting digital holography for imaging of living biospecimens” by Junya Inamoto, Takahito Fukuda, Tomoyoshi Inoue, Kazuki Shimizu, Kenzo Nishio, Peng Xia, Osamu Matoba, and Yasuhiro Awatsuji. https://doi.org/10.1117/1.JBO.25.12.123706
“Imaging performance of microscopy adaptive-optics system using scene-based wavefront sensing” by Yusuke Ashida, Yusuke Honma, Noriaki Miura, Takatoshi Shibuya, Hayao Kikuchi, Yosuke Tamada, Yasuhiro Kamei, Atsushi Matsuda, and Masayuki Hattori. https://doi.org/10.1117/1.JBO.25.12.123707
“Transcutaneous monitoring of hemoglobin derivatives during methemoglobinemia in rats using spectral diffuse reflectance” by Fahima Khatun, Yoshihisa Aizu, and Izumi Nishidate. https://doi.org/10.1117/1.JBO.26.3.033708

We also mention here a BISC-related paper which was published separately as an early-accepted article in October 2020:

“USB capsule endoscope for retrograde imaging of the esophagus” by Ivan Martincek, Peter Banovcin, Matej Goraus, and Martin Duricek. https://doi.org/10.1117/1.JBO.25.10.106002

We believe that this special series on biomedical imaging and sensing is helpful to advance biomedical imaging and sensing techniques. We hope that you agree. The previous two BISC-related special sections were published in JBO Volume 25 Issue 3 and JBO Volume 24 Issue 3; the current

